# Citalopram inhibits platelet function independently of SERT-mediated 5-HT transport

**DOI:** 10.1038/s41598-018-21348-3

**Published:** 2018-02-22

**Authors:** Harvey G. Roweth, Ruoling Yan, Nader H. Bedwani, Alisha Chauhan, Nicole Fowler, Alice H. Watson, Jean-Daniel Malcor, Stewart O. Sage, Gavin E. Jarvis

**Affiliations:** 1 Department of Physiology, Development and Neuroscience, University of Cambridge, Cambridge, U.K.; 2 Department of Biochemistry, University of Cambridge, Cambridge, U.K.

## Abstract

Citalopram prevents serotonin (5-HT) uptake into platelets by blocking the serotonin reuptake transporter (SERT). Although some clinical data suggest that selective serotonin reuptake inhibitors (SSRIs) may affect haemostasis and thrombosis, these poorly-characterised effects are not well understood mechanistically and useful *in vitro* data is limited. We sought to determine whether the inhibitory effects of citalopram on platelets are mediated via its pharmacological inhibition of 5-HT transport. We quantified the inhibitory potency of (*RS*)-, (*R*)- and (*S*)-citalopram on platelet function. If SERT blockade is the primary mechanism for citalopram-mediated platelet inhibition, these potencies should show quantitative congruence with inhibition of 5-HT uptake. Our data show that citalopram inhibits platelet aggregation, adhesion and thromboxane production with no difference in potency between (*R*)- and (*S*)-isomers. By contrast, citalopram had a eudysmic ratio of approximately 17 (*S* > *R*) for SERT blockade. Furthermore, nanomolar concentrations of citalopram inhibited 5-HT uptake into platelets but had no effect on other platelet functions, which were inhibited by micromolar concentrations. Our data indicate that citalopram-induced inhibition of platelets *in vitro* is not mediated by blockade of 5-HT transport. This raises a new question for future investigation: by what mechanism(s) does citalopram inhibit platelets?

## Introduction

Citalopram is a selective serotonin reuptake inhibitor (SSRI) commonly used in the treatment of depression^1^. It inhibits the serotonin transporter (SERT), a member of the solute carrier family of transporters (*SLC6A4*), thereby preventing reuptake of serotonin (5-hydroxytryptamine, 5-HT) into nerve terminals and other cells, including platelets^2^. Most 5-HT in the body is synthesised and located in the gastrointestinal tract^3^. 5-HT released into the portal circulation is metabolised by the liver and transported by SERT into platelets, where it is stored in dense granules, resulting in low plasma concentrations of 5-HT^4^. Platelets are key mediators of both haemostasis and thrombosis, although the role played by 5-HT in these responses is thought to be limited. 5-HT is regarded as a weak platelet agonist, potentiating the activity of other agonists, such as ADP and thromboxane A_2_ (TxA_2_)^5^. Activated platelets release 5-HT, and this may contribute to thrombosis or haemostasis via 5-HT_2A_ receptors on platelets or vascular smooth muscle^6,7^.

Clinical use of SSRIs for depression has been associated with a reduced risk of myocardial infarction^8^ and an increased risk of haemorrhage^9–11^. Such observations combined with SERT expression on platelets have led to suggestions that SSRIs could be used to manage thrombotic disease^12^ via a direct effect on platelets^13–15^. However, other studies have associated SSRI use with an increased risk of myocardial infarction^16,17^, suggesting a more complex picture^18^. Many *ex vivo* studies into the effect of SSRIs on platelet function have been performed. Some reported reduced aggregation^19,20^, and in some cases the effect varied depending on the agonist^2^.

Despite strong circumstantial links between SSRIs, platelets and cardiovascular disease, “*few studies show direct effects in vitro of SSRIs on haemostasis*”^21^. Among those that do, some report inhibitory effects^12,22–24^, some increased aggregation^25^, and others little effect^26^ of SSRIs on platelets.

Depletion of intra-platelet 5-HT stores following prolonged therapy is a mechanism whereby SSRIs could reduce thrombosis^27^. However, direct and rapid effects of SSRIs on platelets *in vitro*^2,22,23^ suggest there may be alternative mechanisms. Citalopram was reported to specifically inhibit collagen-induced aggregation, potentially via a SERT-dependent mechanism^22^. However, the concentration of citalopram required suggested that its mechanism of action was not a direct consequence of SERT inhibition.

The objectives of this study were: (1) to characterise the inhibitory effect of citalopram on platelets; and (2) to determine whether citalopram inhibits platelets in a SERT-dependent manner. Citalopram is a 50:50 racemic (*RS*) mixture of isomers: (*S*)-citalopram is reported to be ~30-fold more potent than (*R*)-citalopram^28^. We used (*RS*)-, (*R*)- and (*S*)-citalopram and compared potencies for inhibition of 5-HT uptake with functional platelet inhibition. We conclude that citalopram does inhibit platelets *in vitro*, although not via inhibition of 5-HT transport. Consequently, alternative mechanisms are necessary to explain the effects of citalopram on platelets.

## Results

### Citalopram inhibits platelet aggregation

Preliminary experiments revealed that citalopram inhibited platelet aggregation at concentrations above 10 µM. Subsequent experiments (using 7 blood donors) were designed to quantify the inhibitory potency of (*RS*)-, (*R*)- and (*S*)-citalopram on collagen- and U46619-induced aggregation. For each donor, full agonist concentration-response curves were obtained at 0, 20, 50 and 100 µM citalopram for collagen, and 0, 50, 100 and 200 µM citalopram for U46619. Collagen and U46619 experiments were conducted on separate occasions.

**Figure 1:**
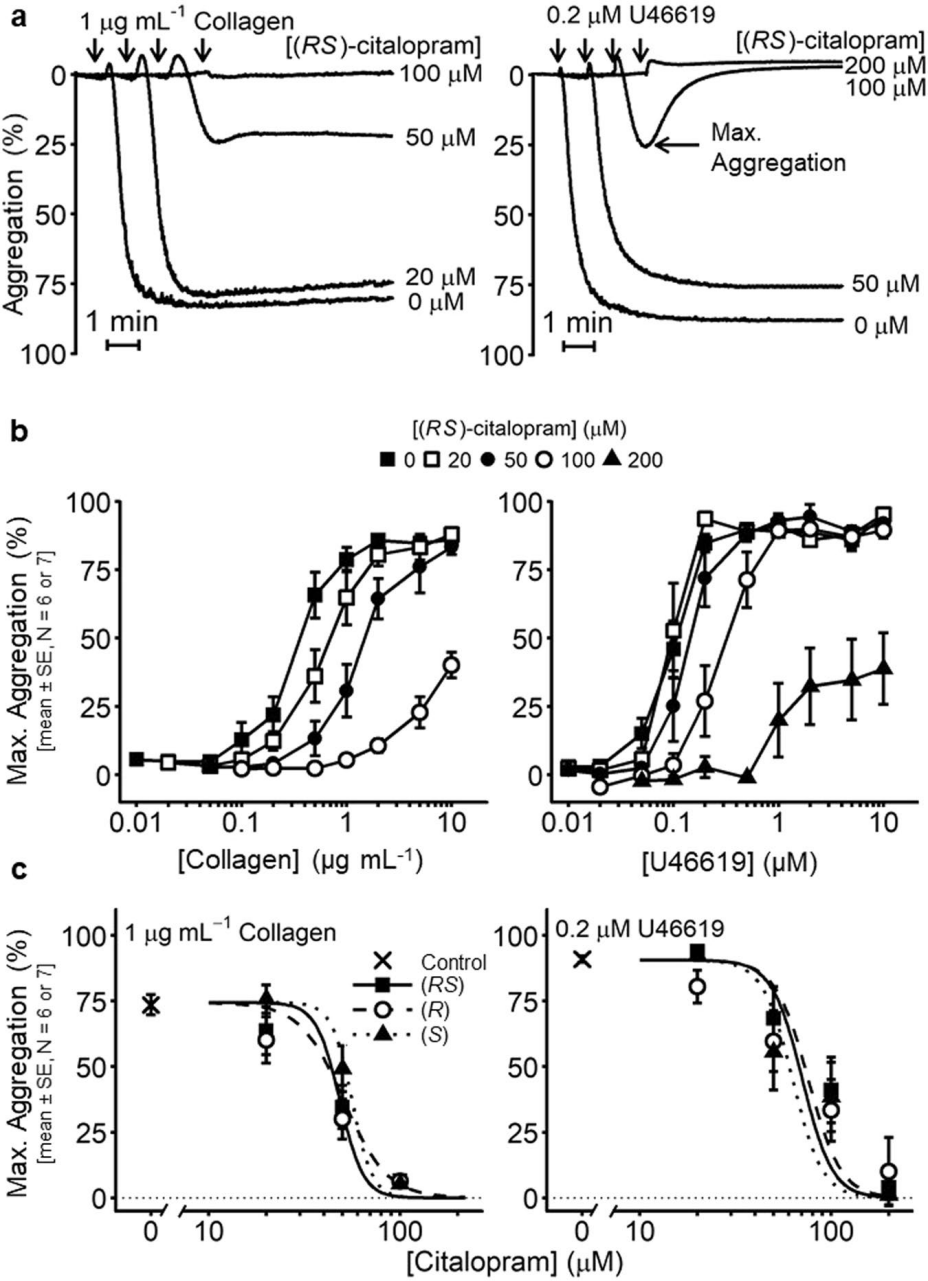
Inhibition by citalopram of platelet aggregation induced by Horm collagen (N = 7 blood donors) and U46619 (N = 6 blood donors). (**a**) Example aggregation traces, illustrating concentration-dependent inhibition of collagen- and U46619-induced platelet aggregation by (*RS*)-citalopram. Arrowheads indicate the addition of agonist. (**b**) Collagen and U46619 concentration-response curves showing inhibition by (*RS*)-citalopram of the maximum extent of aggregation. (**c**) (*RS*)-, (*R*)- and (*S*)-citalopram inhibited the maximum extent of aggregation induced by 1 µg mL^−1^ collagen or 0.2 µM U46619 with similar inhibitory potencies (*pIC*_50_). 3-way ANOVA was performed with IBM SPSS v.23 (Effect 1 (fixed) = citalopram preparation {(*RS*), (*R*), (*S*)}; Effect 2 (fixed) = agonist {collagen, U46619}; Effect 3 (random) = donor {N = 7 (collagen), N = 6 (U46619)}). The results indicated no difference in the *pIC*_50_ of the three citalopram preparations (*P* = 0.95, F = 0.054, degrees of freedom (df) = 2, 27. H_0_: μ_*RS*_ = μ_*R*_ = μ_*S*_; H_1_: μ_*RS*_ ≠ μ_*R*_ ≠ μ_*S*_). Statistical evidence for a difference in the *pIC*_50_ values between collagen and U46619 was weak (*P* = 0.080, F = 3.314, df = 1, 27. H_0_: μ_collagen_ = μ_U46619_; H_1_: μ_collagen_ ≠ μ_U46619_).

Figure 1a shows inhibition of collagen- and U46619-induced aggregation by (*RS*)-citalopram. Figure 1b shows the effect of (*RS*)-citalopram on the maximum extent of aggregation induced by a full range of collagen or U46619 concentrations. Similar inhibition was observed for (*R*)- and (*S*)- citalopram. For each donor, responses to 1 µg mL^−1^ collagen or 0.2 µM U46619 (sub-maximal under control conditions, see Fig. 1b) were fitted to the 4-parameter logistic (4PL) model, with *Max* constrained to zero (Fig. 1c). Citalopram *pIC*_50_ values for both agonists in each donor were obtained and summary results are shown in Table 1. (U46619 data from one experimental day was excluded as the model failed to converge on a meaningful solution). 3-way ANOVA indicated no difference in inhibitory potency between the citalopram isomers (*P* = 0.95). There was a small difference (0.125 ± 0.068) in citalopram *pIC*_50_ values between collagen and U46619 (*P* = 0.08).

**Table 1:** Summary *pIC*_50_ values (mean ± SE (N)) for (*RS*)-, (*R*)- and (*S*)-citalopram on platelet aggregation, static adhesion to collagen, TxB_2_ generation, ATP and ADP release, site-specific protein tyrosine phosphorylation and 5-HT uptake.

Function	Agonist/ligand	*pIC*_50_ (mean ± SE (N))		
		(*RS*)	(*R*)	(*S*)
Aggregation	Collagen	4.31 ± 0.21 (7)	4.29 ± 0.36 (7)	4.25 ± 0.21 (7)
	U46619	4.15 ± 0.27 (6)	4.12 ± 0.22 (6)	4.20 ± 0.29 (6)
Adhesion	Collagen	3.76 ± 0.02 (9)	3.77 ± 0.05 (4)	3.72 ± 0.01 (4)
	CRP	3.78 ± 0.04 (8)	3.81 ± 0.05 (4)	3.74 ± 0.01 (4)
	Fibrinogen	4.00 ± 0.07 (9)	3.92 ± 0.04 (4)	3.92 ± 0.05 (4)
	GFOGER	3.97 ± 0.03 (9)	4.02 ± 0.05 (4)	3.95 ± 0.03 (4)
TxB_2_ synthesis	Collagen	4.77 ± 0.08 (6)	4.68 ± 0.04 (6)	4.70 ± 0.09 (6)
Release (ATP) Release (ADP)	Collagen Collagen	4.54 ± 0.06 (5) 4.51 ± 0.06 (5)		
PLCγ2 (Y1217) SFK (Y416) LAT (Y200)	CRPXL CRPXL CRPXL	4.28 ± 0.03 (5) 4.24 ± 0.03 (4) 3.77 ± 0.03 (5)		
5-HT uptake	5-HT	8.34 ± 0.05 (13)	7.37 ± 0.05 (13)	8.60 ± 0.05 (13)

### Citalopram inhibits static platelet adhesion

**Figure 2:**
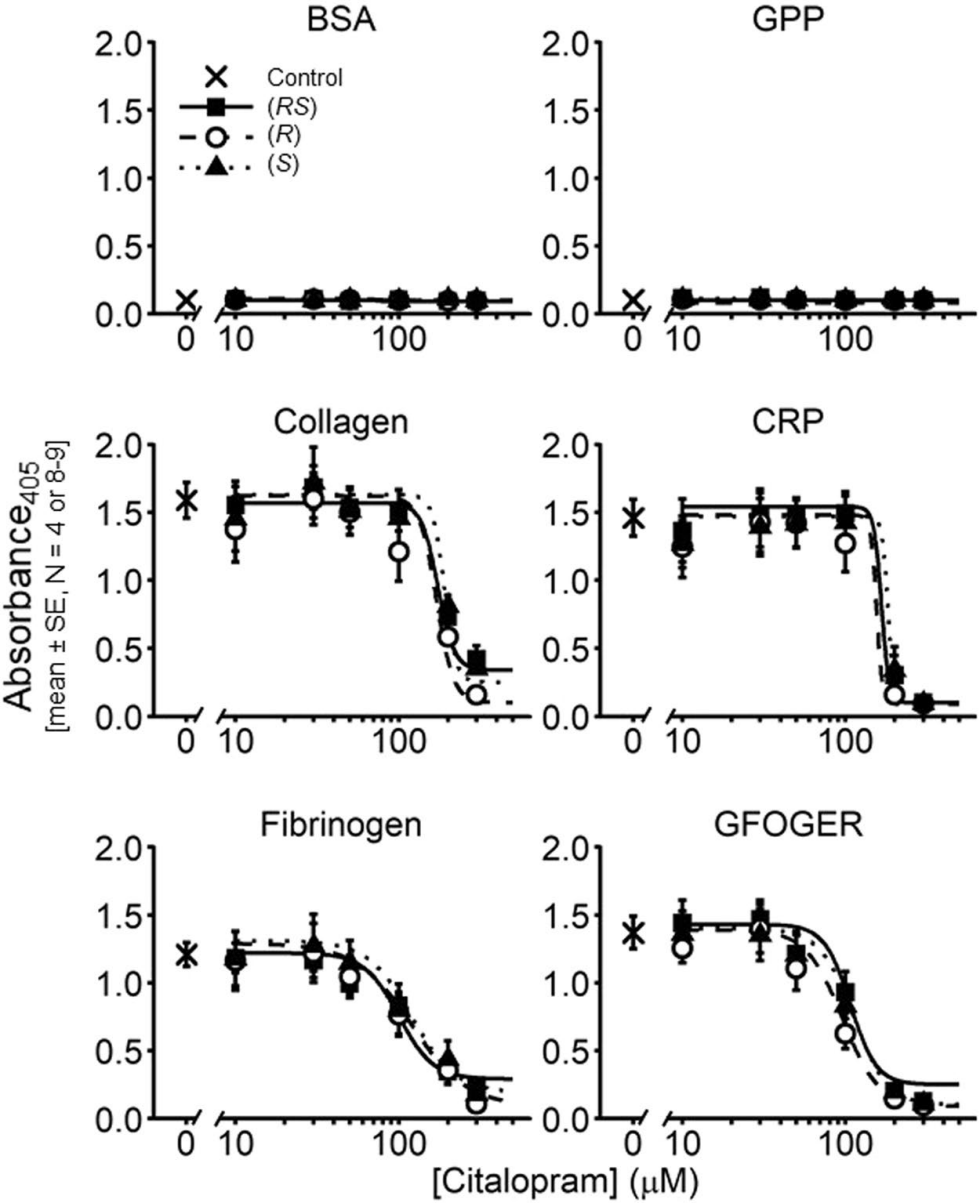
Inhibition by citalopram under static conditions of platelet adhesion to Horm collagen, CRP, fibrinogen and GFOGER. BSA and GPP were used as negative controls. Full concentration-inhibition curves were obtained for (*RS*)-, (*R*)- and (*S*)-citalopram (0–300 µM) for each ligand. 3-way ANOVA was performed with IBM SPSS v.23 (Effect 1 (fixed) = citalopram preparation {(*RS*), (*R*), (*S*)}; Effect 2 (fixed) = ligand {collagen, CRP, fibrinogen, GFOGER}; Effect 3 (random) = donor {N = 8–9 (*RS*) and N = 4 (*R*), (*S*)}). The results indicated no difference in the *pIC*_50_ of the three citalopram preparations (*P* = 0.32, F = 1.18, df = 2, 44. H_0_: μ_*RS*_ = μ_*R*_ = μ_*S*_; H_1_: μ_*RS*_ ≠ μ_*R*_ ≠ μ_*S*_).

The effect of (*RS*)-, (*R*)- and (*S*)-citalopram on the static adhesion of platelets to different adhesive ligands was determined. Full concentration-inhibition curves were constructed using 0, 10, 30, 50, 100, 200 and 300 µM citalopram for four adhesive ligands: (1) collagen; (2) collagen-related peptide (CRP), a selective ligand for the collagen receptor, glycoprotein VI (GPVI); (3) fibrinogen, a ligand for integrin α_IIb_β_3_; and (4) GFOGER, a selective ligand for the collagen receptor, integrin α_2_β_1_^29^. Two negative controls were used: BSA controls for well blocking, and GPP controls for the (GPP)_n_ sequences in GFOGER and for non-specific triple-helical interactions^30^ (Fig. 2). Owing to logistical constraints, all citalopram conditions could not be measured in a single experiment. Hence, in 7 donors (*RS*)-citalopram was tested, in 2 donors (*R*) and (*S*) were tested, and in 2 donors (*RS*)-, (*R*)- and (*S*)-citalopram were tested.

Comparison with the negative controls suggests that citalopram fully inhibited static adhesion of platelets to the adhesive ligands. For each donor and ligand combination, citalopram-induced inhibition of adhesion was fitted to the 4PL model and *pIC*_50_ values obtained (Table 1). 3-way ANOVA indicated no difference in inhibitory potency between the citalopram isomers (*P* = 0.32).

### Citalopram inhibits TxA_2_ synthesis by platelets

**Figure 3:**
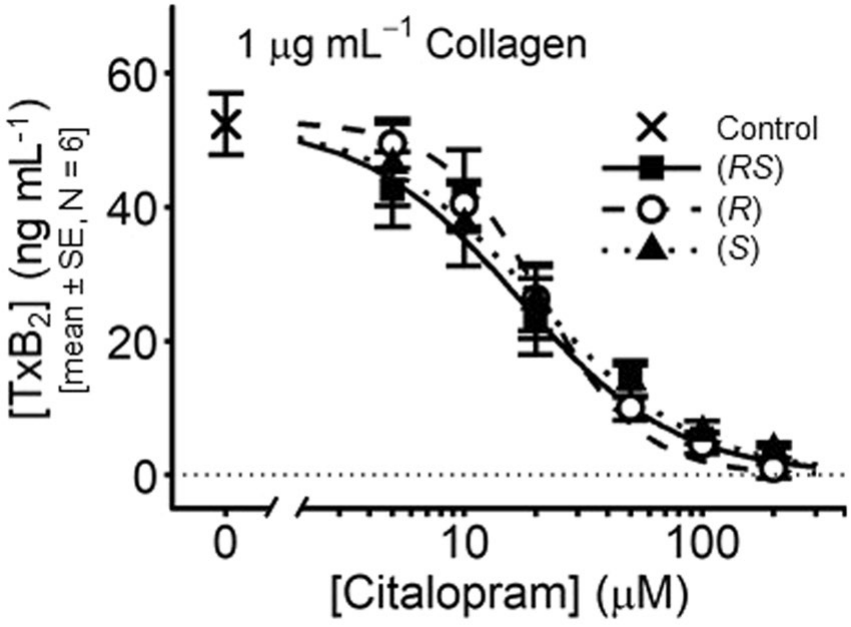
Inhibition by citalopram of TxB_2_ generation. Platelets were stimulated with collagen (1 µg mL^−1^) for 6 min under aggregometry conditions (37 °C, 1,000 rpm) and supernatants isolated. Full concentration-inhibition curves were obtained for (*RS*)-, (*R*)- and (*S*)-citalopram (0–200 µM). 2-way ANOVA was performed with IBM SPSS v.23: (Effect 1 (fixed) = citalopram {(*RS*), (*R*), (*S*)}; Effect 2 (random) = donor {N = 6}). The results indicated no difference in the *pIC*_50_ of the three citalopram preparations (*P* = 0.60, F = 0.54, df = 2, 10. H_0_: μ_*RS*_ = μ_*R*_ = μ_*S*_; H_1_: μ_*RS*_ ≠ μ_*R*_ ≠ μ_*S*_).

TxA_2_ synthesis was indirectly measured by quantifying the generation of its stable metabolite TxB_2_ (N = 6). Washed platelets were pre-treated with a range of (*RS*)-, (*R*)- and (*S*)-citalopram concentrations (0, 5, 10, 20, 50, 100 and 200 µM), before stimulation under aggregometry conditions for 6 min with 1 µg mL^−1^ collagen. *pIC*_50_ values are shown in Table 1. All three citalopram preparations inhibited TxB_2_ generation at micromolar concentrations (Fig. 3). 2-way ANOVA indicated no difference in *pIC*_50_ values between citalopram preparations (*P* = 0.60).

(Aggregometry data obtained from these experiments were independent replicates and reproduced results for inhibition of collagen-induced aggregation: *pIC*_50(*RS*)_ = 4.43 ± 0.08; *pIC*_50(*R*)_ = 4.48 ± 0.07; *pIC*_50(*S*)_ = 4.33 ± 0.06.)

### Citalopram inhibits ATP and ADP release

**Figure 4:**
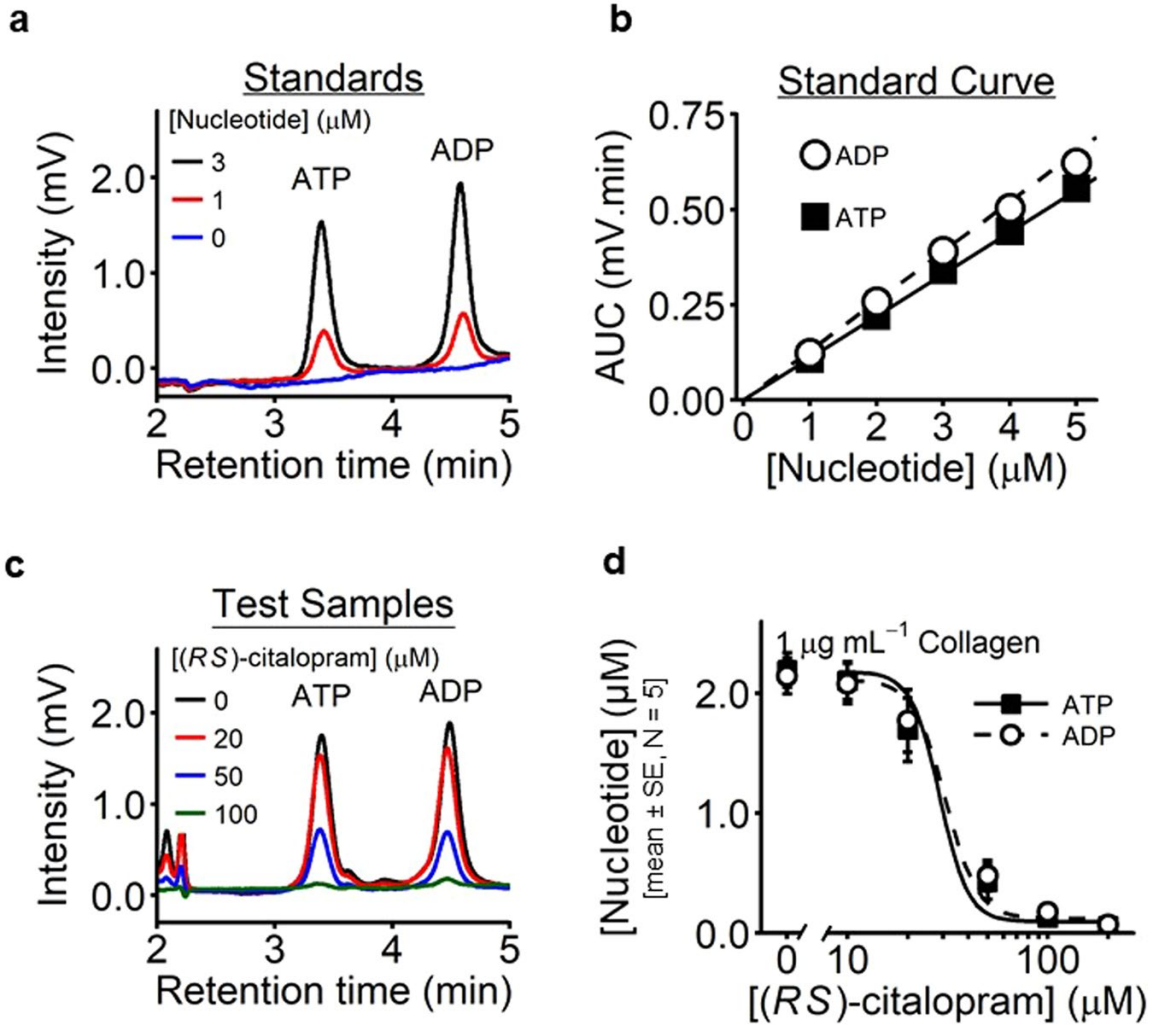
Inhibition by citalopram of collagen-induced release of ATP and ADP from platelets. WP were stimulated with collagen (1 μg mL^−1^) for 6 min under aggregometry conditions (37 °C, 1,000 rpm), supernatants collected, and levels of ATP and ADP quantified using HPLC and absorbance detection at 254 nm (N = 5 blood donors). (**a**) Example HPLC chromatograms, showing known ATP and ADP peaks. (**b**) Peak AUC was plotted against nucleotide concentration to generate standard curves. (**c**) Example HPLC chromatograms from supernatants of collagen-stimulated platelets pre-incubated with (*RS*)-citalopram. (**d**) Concentration-inhibition curves for (*RS*)-citalopram (0–200 µM).

The effect of (*RS*)-citalopram on collagen-induced ATP and ADP release from dense granules was measured (N = 5). Washed platelets were pre-treated with citalopram (0, 10, 20, 50, 100 and 200 µM) before stimulation with collagen (1 µg mL^−1^) for 6 min, under aggregometry conditions. Figure 4a shows high pressure liquid chromatography (HPLC) chromatograms of ATP and ADP, which had retention times of 3.4 and 4.5 min, respectively. Known standards were used to create a calibration curve of peak area under the curve (AUC) versus nucleotide concentration (Fig. 4b), which was used to determine sample nucleotide levels in the presence of varying concentrations of (*RS*)-citalopram (Fig. 4c and d). ATP and ADP release were reduced in a concentration-dependent manner by (*RS*)-citalopram. *pIC*_50_ values were ATP = 4.54 ± 0.06; ADP = 4.51 ± 0.06 (mean ± SE, N = 5) (Table 1).

### Citalopram inhibits tyrosine phosphorylation of PLCγ2, Src family kinases and LAT

**Figure 5:**
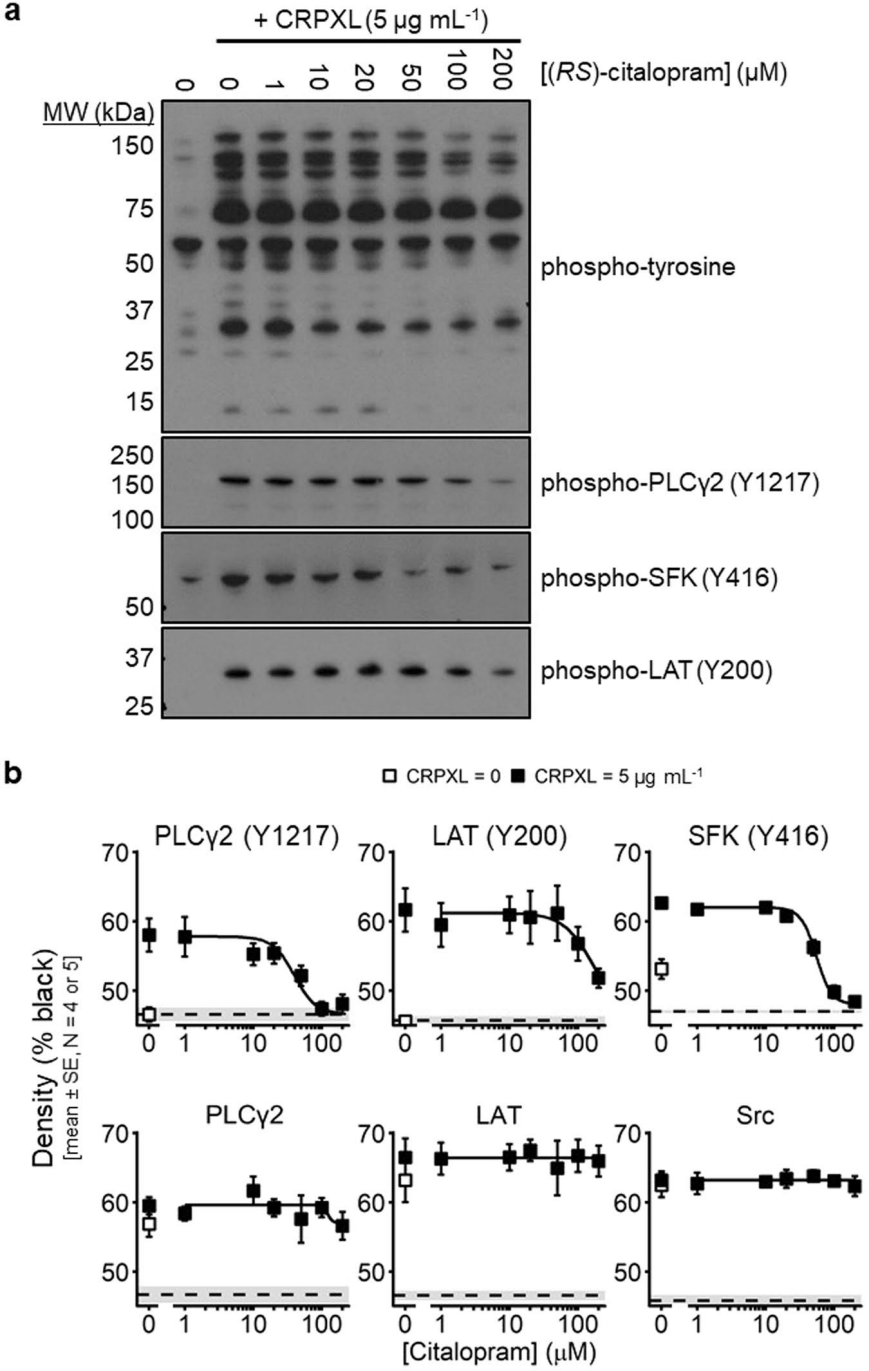
Whole cell tyrosine phosphorylation (4G10) and site-specific phosphorylation of Src family kinases (SFK), LAT and PLCγ2. Platelets were stimulated with 5 µg mL^−1^ CRPXL for 6 min under aggregometry conditions in the presence of GR 144053 (2 µM) to prevent aggregation and facilitate solubilisation of protein. (**a**) Images from non-blinded pilot experiments show the effect of (*RS*)-citalopram on whole cell tyrosine phosphorylation and site-specific phosphorylation of PLCγ2, SFK and LAT. The whole cell tyrosine phosphorylation blot is one of two repeats. The phospho-specific blots are from additional pilot experiments. Images have been cropped from four different blots. Images of full-length blots from these experiments are presented in Supplementary Fig. S1. (**b**) Densitometric quantification from blinded and randomised experiments measuring site-specific phosphorylation of PLCγ2 (N = 5), SFK (N = 4) and LAT (N = 5) in resting or stimulated platelets. Blots are shown in Supplementary Figs. S2 and S3. Dashed lines (mean) and grey area (±SE) indicate background signal on the developed X-ray films. Lower panels represent total protein levels from the same samples. Concentration-responses were modelled using NONMEM 7.3 to accommodate random between experiment variability often associated with Western Blotting. Confirmation of the phosphorylation response was performed using likelihood ratio tests by comparing the following statistical hypotheses: H_0_: *Max* = *Min* and H_1_: *Max* ≠ *Min*. Results were: phospho-PLC (Y1217), *P* = 2 × 10^−13^ (χ^2^ = 65.1, df = 4); phospho-SFK (Y416), *P* = 7 × 10^−25^ (χ^2^ = 115.7, df = 3); phospho-LAT (Y200), *P* = 4 × 10^−10^ (χ^2^ = 49.8, df = 4). Evaluation of total protein levels was performed by comparing the hypotheses: H_0:_ *Max* = *Min* = *Basal*; and H_1_: *Max* ≠ *Min* ≠ *Basal* (*Basal* = signal when CRP = 0 µg ml^−1^). Results were: PLC, *P* = 0.30 (χ^2^ = 6.04, df = 5); Src, *P* = 0.84 (χ^2^ = 1.39, df = 4); LAT, *P* = 0.30 (χ^2^ = 4.89, df = 4).

Preliminary non-blinded experiments (Fig. 5a, Supplementary Fig. S1) indicated that citalopram reduced whole cell tyrosine phosphorylation, including the 10–15 kDa Fc receptor gamma chain (FcRγ), and site-specific tyrosine phosphorylation of Src family kinases (SFK) (Y416), Linker of Activated T cells (LAT) (Y200) and phospholipase Cγ2 (PLCγ2) (Y1217), all on residues associated with their activation. SFK, LAT and PLCγ2 are situated respectively at proximal, intermediate and distal positions in the GPVI signalling pathway, with PLCγ2 mediating the release of calcium from intracellular stores into the cytosol. Protein samples from subsequent experiments were randomly loaded into pre-cast gels in a blinded fashion and membranes probed for PLCγ2 (N = 5) and LAT (N = 5) (Supplementary Fig. S2) and SFK (N = 4) (Supplementary Fig. S3). In each experiment, basal phosphorylation (cross-linked collagen-related peptide (CRPXL) = 0) and concentration-inhibition curves (CRPXL = 5 µg mL^−1^; (*RS*)-citalopram = 0, 1, 10, 20, 50, 100 and 200 µM) were determined and analysed using non-linear mixed effects modelling (Fig. 5b). *pIC*_50_ values are in Table 1. (*RS*)-citalopram inhibited CRPXL-induced tyrosine phosphorylation of PLCγ2 (*P* = 2 × 10^−13^), SFK (*P* = 7 × 10^−25^) and LAT (*P* = 4 × 10^−10^). No phosphorylated PLCγ2 or LAT was detected in unstimulated platelets, although there was a basal signal for phosphorylated SFK (Fig. 5b). There was no relationship between CRPXL or (*RS*)-citalopram and total levels of PLCγ2 (*P* = 0.30), Src (*P* = 0.84) or LAT (*P* = 0.30) (Fig. 5b).

### Citalopram blocks uptake of 5-HT into platelets

Experiments were designed to measure the inhibitory potency (*pIC*_50_) of (*RS*)-, (*R*)- and (*S*)-citalopram on 5-HT uptake into platelets. 5-HT (1 µM) was added to washed platelets (2.00 × 10^8^ mL^−1^) and its extracellular concentration measured over time. The decline in concentration followed a first order pattern (see Eq. 2) and hence each kinetic profile (n = 6–12 time points per profile) yielded a single rate constant for uptake (*k*_*u*_). The rate constant is the probability of 5-HT uptake per unit time and is therefore directly proportional to the level of SERT activity. Consequently, a 50% reduction in *k*_*u*_ from baseline indicated 50% citalopram binding.

**Figure 6:**
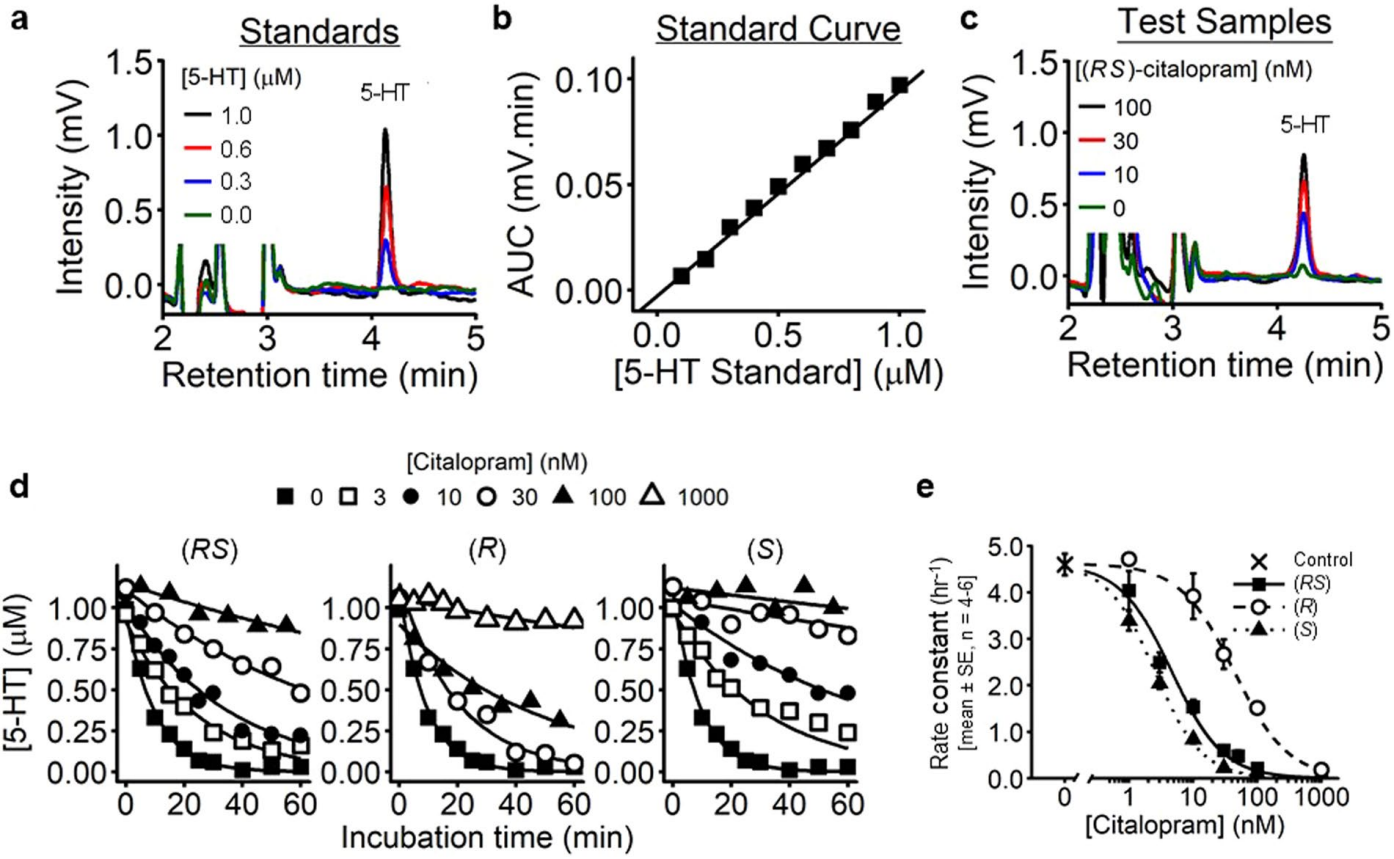
Inhibition of 5-HT uptake into platelets by citalopram. (**a**) Example HPLC chromatograms, showing peaks for known concentrations of 5-HT. (**b**) Peak AUCs, detected at 276 nm, were plotted against standard 5-HT concentrations to construct a calibration curve. (**c**) Example chromatograms used to quantify the supernatant 5-HT concentration 30 min after the addition of 1 µM 5-HT to WP. (**d**) Profiles showing the reduction in supernatant 5-HT over time. Uptake was blocked by increasing concentrations of (*RS*)-, (*R*)- and (*S*)-citalopram. Kinetic profiles (each with 6–12 time points) were used to determine rate constants of uptake (*k*_*u*_) under differing citalopram conditions. (**e**) The concentration-dependent effect of (*RS*)-, (*R*)- and (*S*)-citalopram (0–1,000 nM) on *k*_*u*_ was fitted to the 4PL model using NONMEM 7.3 and nested models compared using Likelihood Ratio Tests. Each citalopram preparation abolished 5-HT uptake in a manner consistent with non-cooperative 1:1 binding (*P* = 0.099, LRT, χ^2^ = 10.7, df = 6. H_0_: *n*_*H*_ = 1, *Max* = 0; H_0_: *n*_*H*_ ≠ 1, *Max* ≠ 0 for each preparations of citalopram. N = 13 separate experiments).

Figure 6a and b show HPLC chromatogram peaks from known 5-HT concentrations (retention time of 4.25 min) and a standard curve for 5-HT from one experimental day, respectively. Figure 6c shows a test HPLC chromatogram from platelet supernatants. The concentrations of supernatant 5-HT were derived from the peak AUC and the standard curve.

Rate constants (*k*_*u*_) were obtained for (*RS*)-, (*R*)- and (*S*)*-*citalopram over a range of concentrations, which varied depending on the citalopram isomer (Fig. 6d). Owing to logistical constraints, it was not possible to collect data for all conditions on each experimental day. In total, 76 rate constants from 11 donors were obtained on 14 separate occasions. Data from one day/donor (comprising 4 rate constants) were excluded from the final analysis as a hyper-functional outlier, lying 7 SDs beyond the basal range as defined by all the other data. All remaining data for the three citalopram inhibition curves were modelled simultaneously to the 4PL using NONMEM 7.3, allowing estimation of the random variance in basal activity between different experimental days.

Basal *k*_*u*_ (mean ± SE) was 4.60 ± 0.23 hr^−1^ and the inter-experimental standard deviation ± SE was 0.75 ± 0.17 hr^−1^. Citalopram abolished uptake (*Max*_(*RS*)_ = 0.09 ± 0.04; *Max*_(*R*)_ = −0.02 ± 0.11; *Max*_(*S*)_ = 0.01 ± 0.05) with Hill coefficients close to unity (*n*_*H*(*RS*)_ = 1.08 ± 0.09; *n*_*H*(*R*)_ = 1.03 ± 0.12; *n*_*H*(*S*)_ = 1.18 ± 0.12) (*P* = 0.099: see Fig. 6e for further detail). This is consistent with non-cooperative 1:1 binding of citalopram to SERT, thereby causing complete blockade of 5-HT transport. The isomers had different inhibitory potencies: (*S*)-citalopram (*pIC*_50(*S*)_ = 8.60 ± 0.05) was approximately 17-fold more potent than (*R*)-citalopram (*pIC*_50(*R*)_ = 7.37 ± 0.05) and (*RS*)-citalopram (*pIC*_50(*RS*)_ = 8.34 ± 0.05) was approximately 1.8-fold less potent than (*S*)-citalopram, suggesting that the inhibitory activity of (*RS*)-citalopram was predominantly caused by (*S*)-citalopram. The mechanistic significance of these data is discussed below.

### Cytotoxic effects of citalopram on platelets

**Figure 7:**
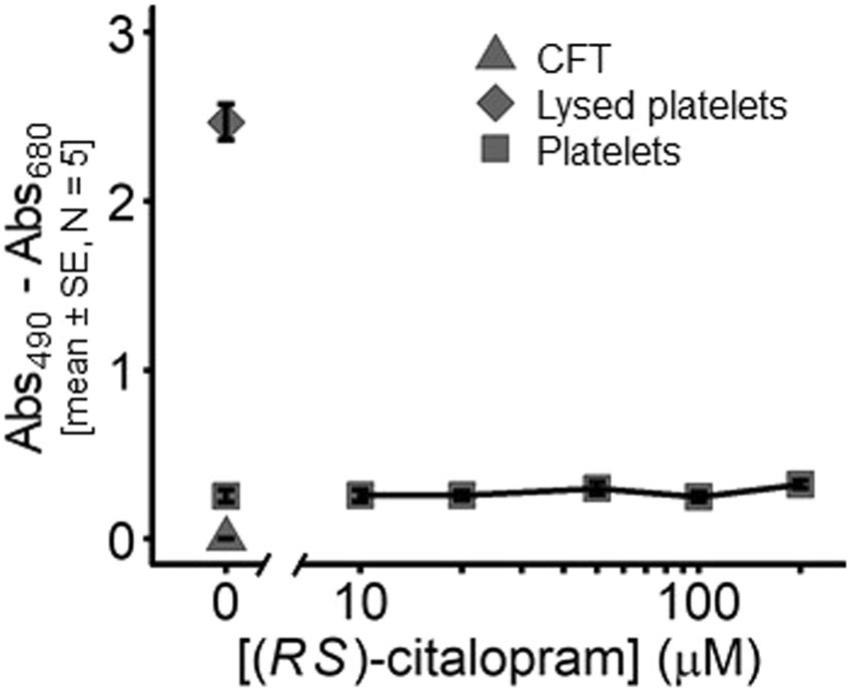
Micromolar concentrations of citalopram do not cause cytotoxicity, as indicated by LDH release from platelets. Platelets were incubated for 10 min with (*RS*)-citalopram (0–200 µM). Calcium-free Tyrodes (CFT) and lysed platelets were used as negative and positive controls, respectively (N = 5 blood donors).

An experiment (N = 5 donors) was designed to determine whether the citalopram concentrations used throughout this study were cytotoxic to platelets. Washed platelets (2.00 × 10^8^ mL^−1^) were incubated for 10 min with citalopram (0, 10, 20, 50, 100 and 200 μM) and levels of supernatant lactate dehydrogenase (LDH) quantified. Citalopram did not cause LDH release at any tested concentration (Fig. 7).

## Discussion

This is not the first time that citalopram or other SSRIs have been shown to inhibit platelet function *in vitro*^2,22,23^. However, implicit in some previous studies is the mechanistic conclusion that functional inhibition of platelets by SSRIs is due to SERT inhibition. The expression on platelets of SERT^31^, the molecular target of SSRIs, lends credibility to this conclusion. The principal objective of our study was to determine whether functional inhibition of platelets by citalopram could be mechanistically linked with SERT blockade.

**Figure 8:**
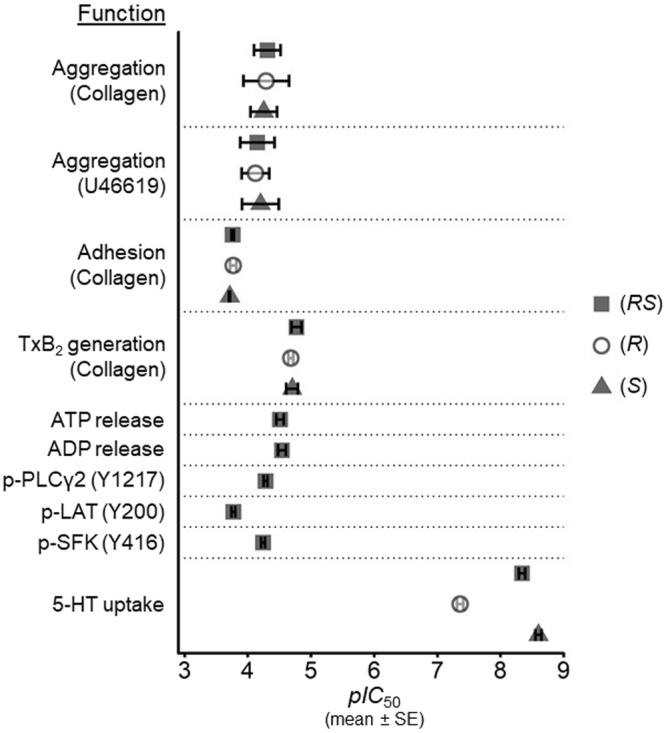
Summary results, showing the difference in *pIC*_50_ values (mean ± SE) between the inhibition of 5-HT uptake by citalopram isomers (Fig. 6) and their inhibition of platelet aggregation (Fig. 1), adhesion to Horm collagen (Fig. 2), TxB_2_ generation (Fig. 3), nucleotide release from dense granules (Fig. 4) and the site-specific phosphorylation of Src family kinases (SFK), LAT and PLCγ2 (Fig. 5).

Citalopram is a racemate, comprising low (*R*) and high (*S*) potency isomers. We quantified the inhibitory potency of the isomers and the racemate against different platelet functions and 5-HT uptake using the same preparation of platelets to enable direct comparison of the results (Fig. 8 and Table 1). Our data confirm the difference in potency between (*R*)- and (*S*)-isomers in inhibiting 5-HT uptake^28^. By contrast, there was no difference in the potencies of the isomers in inhibiting platelet aggregation, adhesion or TxA_2_ synthesis. Furthermore, the concentrations required to inhibit all platelet functions were substantially in excess of those required to inhibit SERT. At concentrations less than 1 µM, 5-HT uptake was completely blocked by both (*R*)- and (*S*)-isomers, whereas there was no indication of functional inhibition. High concentrations of citalopram that blocked platelet function did not cause cell destruction (Fig. 7), suggesting that the observed inhibition is not a non-specific toxic effect. We therefore conclude that platelet inhibition by citalopram *in vitro* is not dependent on the inhibition of SERT-mediated 5-HT uptake, and that other mechanisms must be identified to explain these antiplatelet effects of citalopram.

Our results are partly consistent with those from earlier studies^22,23^. Contrary to previous claims^22^, we demonstrate that platelet inhibition by citalopram is not collagen-specific, since it clearly inhibits U46619-induced aggregation. Furthermore, their observation^22^ that citalopram did not inhibit collagen-induced PAC-1 binding to integrin α_IIb_β_3_ was surprising, given that citalopram inhibited collagen-induced aggregation, and especially since it did block convulxin-induced PAC-1 binding^22^. Tseng *et al*. suggested that the GPVI pathway was blocked, but that collagen might act via a citalopram-insensitive pathway, for example, α_2_β_1_ signalling. By contrast, our results with collagen and the GPVI-selective ligand CRPXL provide no reason to suppose that inhibition of collagen-induced responses by citalopram is mediated exclusively via disruption of α_2_β_1_ signalling.

It has previously been reported that citalopram is an allosteric serotonin reuptake inhibitor^32^. A complex mechanism of action has been proposed with two distinct binding loci, a primary and an allosteric site, on SERT. It has been suggested that binding of either (*R*)- or (*S*)-isomers to an allosteric site has differential effects on the primary binding site^33^, thus providing a mechanistic explanation for the more rapid onset of action of (*S*)-citalopram in animal models of depression^34^, and greater potency in clinical trials^35^. By contrast, our data are consistent with a simple mechanism of action in which both (*R*)- and (*S*)-isomers bind to the same site on SERT but with differing affinities. Hill coefficients of unity and complete inhibition of 5-HT uptake suggest that non-cooperative binding of citalopram completely blocks SERT. Inhibition caused by simultaneous application of two different drugs differs when those drugs either compete for the same site (syntopic model) or bind to separate sites (allotopic model)^36^. We fitted our 5-HT uptake data to both syntopic and allotopic models using NONMEM: in both cases the number of estimated parameters was the same, but the predicted effect of the racemic mixture differed between the two models. The difference in the Bayes Information Criterion^37^ for the two models was 14.0, with the syntopic model having the lower value. Following the convention of Kass and Raftery^38^, this constitutes “very strong” evidence in favour of the syntopic model. Taken altogether, our findings suggest that at concentrations of up to 1 µM, inhibition of 5-HT uptake by citalopram is consistent with a simple non-cooperative binding model in which the (*R*)- and (*S*)-isomers bind to the same site but with different affinities.

Both (*R*)- and (*S*)-citalopram slow the dissociation of bound [^3^H]-(*S*)-citalopram from SERT at concentrations from 1–200 µM^39,40^, levels in excess of those required to block 5-HT uptake. A daily dose of 30–60 mg citalopram gives steady-state plasma concentrations of 120–600 nM^41^ and approximately 50–80% of citalopram is bound to plasma proteins^41,42^. Therefore, clinical doses of either (*RS*)- or (*S*)-citalopram generate plasma concentrations that will substantially block 5-HT uptake, but will have neither the allosteric effect on SERT, nor the antiplatelet effects reported in our study. It is therefore unlikely that a typical therapeutic regime of citalopram will have any anti-haemostatic or anti-thrombotic effect as a consequence of the functional inhibitory effects we have described. Long-term treatment with citalopram depletes platelet 5-HT^2,27^ and this may provide an alternative mechanism for any observed clinical effects on thrombosis and haemostasis.

Although there is no direct relationship between blockade of 5-HT uptake and platelet inhibition, it remains possible that SERT blockade is permissive for additional mechanisms that citalopram may exert at higher concentrations. However, we do not consider this to be likely and have no evidence that positively supports this hypothesis. Interestingly, the range of citalopram concentrations that exert an allosteric effect on SERT, whilst not of clinical significance, is similar to the range within which we observe platelet functional inhibition.

Other mechanistic associations with SERT have been described that may contribute to the functional effects of citalopram, including P2Y_12_ signalling^23^, 5-HT_2A_ receptor desensitisation^43^ and integrin α_IIb_β_3_^24^. In the case of α_IIb_β_3_, co-expression in HEK293 cells of human SERT with either Leu33 or Pro33 variants of the β_3_ integrin resulted in differential surface expression of SERT and a consequent effect on 5-HT transport. Similarly, platelets from Leu33 and Pro33 homozygotes also showed differential sub-cellular distribution of SERT with lower relative surface expression in non-mutant Leu33 individuals^24^. This, and their observation that fibrinogen-bound platelets had a greater rate of 5-HT uptake suggest a functional interaction between SERT and α_IIb_β_3_, which may provide a mechanistic basis for the actions of citalopram in platelets. However, in a study on SERT-deficient murine platelets, a physical interaction between SERT and β_3_ integrin was found to be not essential for inside-out integrin activation^44^. These conflicting findings bring into question the significance of any association between SERT and α_IIb_β_3_, at least in mouse platelets, and therefore do not provide a clear mechanistic basis for the actions of citalopram.

Citalopram has been reported to have SERT-independent effects in the nervous system via an action on sigma-1 (σ1) receptors^45^. Other SSRIs also have affinity for σ1 receptors^46^ and peptide sequences from the σ1 receptor have been identified in platelets^47^. These observations suggest that the σ1 receptor may be a target mediating the SERT-independent effects of citalopram in platelets. However, Bonnin *et al*. observed no effect with (*S*)-citalopram and concluded that SERT-independent action was mediated by the (*R*)-isomer. This is clearly not consistent with our observations, and the reported K_i_ of 0.3 µM of citalopram for σ1 receptors^46^ further undermines the hypothesis that σ1 may be mediating the effects of citalopram in platelets.

Although our study was designed principally to address the question of the role of SERT, there are clues in the data that point to potential novel mechanisms of action. Figure 1b and c clearly indicate that citalopram is more potent at inhibiting collagen than U46619-induced aggregation. The effect of 20 µM citalopram may therefore be on a collagen-specific mechanism. The effect of 20 and 50 µM citalopram on collagen looks competitive, suggesting an action at or close to the level of the receptor, GPVI. Inhibition of phosphorylation of GPVI signalling proteins including PLCγ2, LAT, SFKs and FcRγ chain also supports this hypothesis. At 100 µM citalopram, inhibition of U46619 is clear and of collagen is exaggerated (Fig. 1b), suggesting a distinct mechanism. The signalling pathways of GPVI and the thromboxane receptor converge with an elevation of intracellular calcium. The data may therefore indicate an effect of citalopram on either calcium signalling or a process further downstream at concentrations higher than those required to inhibit GPVI signalling. The collapse of the U46619 response at 200 µM is also consistent with this hypothesis. Preliminary data that support these hypotheses have recently been presented^48^.

The platelet inhibitory effects of citalopram occur at relatively high concentrations, and it is therefore unlikely that these effects could be exploited clinically. However, after identifying a mechanism, citalopram could serve as a chemical starting point for the development of more potent and selective inhibitors of that mechanism, just as ATP, a clinically impractical, non-selective, low potency inhibitor of the P_*2T*_ receptor^49,50^ became the starting point for a drug discovery project that produced the P2Y_12_ antagonist ticagrelor^51^. Hence, the study of the pharmacology of low potency compounds can be both biologically enlightening and practically beneficial.

In summary, we have shown that inhibition of SERT-dependent 5-HT uptake by citalopram does not correlate with inhibition of platelet function *in vitro*. Furthermore, the mechanisms that underlie citalopram-induced platelet inhibition *in vitro* are unlikely to influence haemostasis and thrombosis *in vivo*. Long-term citalopram treatment, depletion of platelet 5-HT and disruption of 5-HT-mediated effects on blood vessels may do so. We therefore conclude that the antiplatelet effects of citalopram observed at micromolar concentrations *in vitro* must be mediated by pharmacological mechanisms distinct from SERT inhibition and the blockade of 5-HT uptake. Further investigations are ongoing to identify potential molecular targets in platelets responsible for these effects of citalopram.

## Materials and Methods

### Materials

Fibrinogen, *p*-nitrophenyl phosphate, prostaglandin E_1_, indomethacin, U46619 (a TxA_2_ mimetic), citric acid, EDTA, trisodium citrate, ATP, ADP and potassium phosphate dibasic were from Sigma (Poole, U.K.). EGTA was from Calbiochem (Nottingham, U.K.). (*RS*)-citalopram and (*S*)-citalopram were from Cambridge Bioscience (Cambridge, U.K.). (*R*)-citalopram was from Insight Biotechnology (Wembley, U.K.). Acetonitrile and potassium phosphate monobasic were from Fisher Scientific (Loughborough, U.K.). 5-HT was from Alfa Aesar (Lancashire, U.K.). Horm collagen was from Takeda (Linz, Austria). BSA was from G.E. Healthcare (Buckinghamshire, U.K.). GR 144053 was from Tocris Bioscience (Bristol, U.K.). The following synthetic collagen-mimetic peptides were synthesised in the laboratory of Professor Richard Farndale (University of Cambridge, U.K.): collagen-related peptide (CRP: sequence = GCO(GPO)_10_GCOG-NH_2_, O = hydroxyproline); cross-linked CRP (CRPXL); GPP (sequence = GPC(GPP)_10_GPC-NH_2_); and GFOGER (sequence = GPC(GPP)_5_GFOGER(GPP)_5_GPC-NH_2_)^30^. Antibodies against phospho-SFK (Y416) (#2101 S)^52^, Src (#2108 S)^52^ and phospho-PLCγ2 (Y1217) (#3871)^53^ were from Cell Signalling Technology (Danvers, MA, U.S.A.). Antibodies against LAT (#06-807)^54^ and phospho-tyrosine (4G10) (#05-321)^55^ were from Millipore (Watford, U.K.). Antibodies against phospho-LAT (Y200) (#ab68139)^56^ were from Abcam (Cambridge, U.K.). Antibodies against PLCγ2 (#sc-407)^57^ were from Santa Cruz (Wembley, U.K.). HRP-conjugated mouse (#P0447)^58^ and rabbit (#P0448)^58^ secondary antibodies were from Dako (Ely, U.K.).

### Washed platelet preparation

Donation of fresh blood from healthy, consenting human volunteers was approved by the University of Cambridge Human Biology Research Ethics Committee (Ref: HBREC.2015.18). Prior to any blood donation, informed consent was obtained from each blood donor. The consent form was signed by both the donor and one of the project supervisors (G.E.J. or S.O.S.). A fresh consent form is signed annually. On the occasion of each donation of blood, a donation record form was signed by both the donor and the phlebotomist (G.E.J., S.O.S. or H.G.R.). Collection of blood from donors, its use and subsequent disposal were performed in accordance with relevant guidelines and regulations.

Blood was drawn into syringes containing trisodium citrate (final concentration of 11 mM) and centrifuged (500 × g, 5 min) to obtain platelet-rich plasma. Following addition of prostaglandin E_1_ (final concentration of 1 μM), platelet-rich plasma was centrifuged (900 × g, 15 min) and the resulting platelet pellet re-suspended in a modified calcium-free Tyrode’s buffer (CFT; 137 mM NaCl, 11.9 mM NaHCO_3_, 0.4 mM NaH_2_PO_4_, 2.7 mM KCl, 1.1 mM MgCl_2_, 5.6 mM glucose; pH = 7.4). Platelet counts were adjusted using a Z2 COULTER COUNTER (Beckman Coulter, High Wycombe, U.K.). Platelets were usually pre-treated with (*RS*)-, (*R*)- or (*S*)-citalopram for approximately 3–5 mins before each experiment. For the aggregation experiments, citalopram was simultaneously added to all washed platelet samples in advance of the measurements, and therefore some samples were incubated for longer periods of time, up to 3 hours. Preliminary data gave no indication that inhibitory effect of citalopram was time-dependent within this time-scale.

### Platelet aggregometry

Platelet aggregation was measured by turbidimetric aggregometry^59,60^, using two Aggregation Remote Analyser Modules (AggRAM) with HemoRAM software (v1.2) (Helena Biosciences, Newcastle, U.K.). Washed platelets (WP) (247.5 µL, 2.00 × 10^8^ mL^−1^) were aliquoted into glass cuvettes containing magnetic stir bars. 2.5 µL agonist was added and aggregation recorded at 37 °C with a stir speed of 1,000 rpm. The maximum extent of aggregation over 6 min was determined^60^.

### Static platelet adhesion

Platelet adhesion under static conditions was measured by detecting acid phosphatase levels in platelet lysates^61,62^. 100 μL of adhesive ligand (10 μg mL^−1^ in saline or 0.01 M acetic acid) was incubated in Immulon-2HB 96-well flat bottom plates overnight at 4 °C. Ligands were randomly assigned to different rows for each experiment. Excess ligand was discarded, and the wells blocked with 175 μL BSA (5% [w/v] in CFT) for 1 hour. Wells were then washed 3 times with BSA (0.1% [w/v] in CFT). 50 µL WP (1.25 × 10^8^ mL^−1^) were added to wells and left at room temperature (RT) for 1 hour. WP containing different drug concentrations were randomly assigned to different columns for each experiment. Excess WP were discarded and the wells washed a further three times, followed by addition to each well of 150 μL citrate lysis buffer (3.53 mM *p*-nitrophenyl phosphate, 71.4 mM trisodium citrate, 28.55 mM citric acid, 0.1% [v/v] Triton X-100, pH = 5.4). After 1 hour incubation at RT, 100 μL of 2 M NaOH was added to each well and absorbance measured at 405 nm using an ELx808 plate reader (Biotek, Swindon, U.K.).

### Thromboxane A_2_ synthesis

On activation, platelets produce the pro-aggregatory, vasoconstrictor TxA_2_. Inhibition of TxA_2_ synthesis is thought to underlie the anti-thrombotic effects of aspirin. TxA_2_ is rapidly hydrolysed in aqueous media to TxB_2_, which is stable. We measured TxB_2_ generation using the competitive Cayman TxB_2_ Express ELISA kit (Cambridge Bioscience, Cambridge, U.K.).

WP (247.5 µL, 2.00 × 10^8^ mL^−1^) were activated as for aggregometry. 6 min after agonist addition, the STOP solution was added, samples were immediately centrifuged (12,000 rpm, 2 min) and the supernatants frozen at −80 °C. Samples were later thawed and diluted 1:40 or 1:200 in ELISA buffer (100 mM phosphate, 0.1% [w/v] BSA, 400 mM NaCl, 1 mM EDTA, 0.01% [w/v] sodium azide) and 50 µL added to wells of a polyclonal goat anti-mouse IgG-coated plate. 50 µL of TxB_2_ standards were aliquoted to determine the relationship between absorbance and TxB_2_ concentration. 50 µL of TxB_2_-acetylcholinesterase tracer and 50 µL of anti-TxB_2_ monoclonal antibody were added to each well and incubated for 2 hours at RT. Wells were washed four times with wash buffer and incubated with 200 µL Ellman’s Reagent under dark conditions. Absorbance at 405 nm was measured periodically using a Tecan Sunrise absorbance microplate reader (Tecan U.K., Reading, U.K.), until the absorbances of wells containing no sample TxB_2_ were in the range of 0.3–1.5 absorbance units (A.U.).

Absorbance values for TxB_2_ standards were fitted to a 4PL model and test sample values were converted to TxB_2_ concentrations using the inverse function.

### Platelet nucleotide release

WP (247.5 µL, 2.00 × 10^8^ mL^−1^) were activated by the addition of 2.5 µL of agonist in the AggRAM modules (37 °C, 1,000 rpm). 50 µL of a STOP solution (final concentration = 5 mM EGTA, 16.6 μM indomethacin) was added after 6 min to minimise further nucleotide release. Samples were immediately centrifuged (1 min, 13,000 rpm) and supernatants collected and frozen.

Supernatant concentrations of ATP and ADP were quantified using HPLC with a Waters 2795 Separations Module and a Waters 2847 Dual λ Absorbance Detector (Waters, Hertfordshire, U.K.). Nucleotides were separated using a gradient method on a reversed phase C18 column with polar endcapping to tolerate a 100% aqueous phase (Synergi Hydro-RP: 250 × 4.6 mm, 4 μm beads, 80 Å pore size) (product code 00G-4375-E0, Phenomenex, Cheshire, U.K.). Two mobile phases were used, a phosphate buffer (2.2 mM K_2_HPO_4_, 47.8 mM KH_2_PO_4_; pH = 5.45) and acetonitrile. Nucleotides were separated over 6 min at a constant flow rate of 1.7 mL min^−1^. Details of the gradient method are in Supplementary Table S1. Peaks for ATP and ADP were detected at 254 nm, with retention times of 3.4 and 4.5 min, respectively. Chromatograms were analysed using N2000 Chromatography Data System (Tianjin University, China). Nucleotide levels were quantified as AUC of the peaks, and converted into concentrations using linear ATP and ADP standard curves, prepared on each analytical day.

### Protein tyrosine phosphorylation

WP (247.5 µL, 2.00 × 10^8^ mL^−1^) were stimulated with CRPXL (5 µg mL^−1^) in AggRAM modules (37 °C, 1,000 rpm) for 6 min in the presence of GR 144053 (2 µM), an integrin α_IIb_β_3_ antagonist, to prevent aggregation. WP were diluted 5:1 in Laemmli sample buffer (final concentrations = 25 mM Tris HCl, 0.4% [v/v] glycerol, 0.8% [w/v] SDS, 1% [v/v] mercaptoethanol, 0.01% [w/v] brilliant blue) and heated for 10 min at approximately 100 °C. Molecular weight markers (Precision plus protein standards, Bio-Rad Laboratories, Hertfordshire, U.K.) were added to wells 1 and 10, and protein samples randomly assigned and added in a blinded fashion to wells 2–9 of 10 well 4–12% pre-cast NuPage Bis-Tris gels (Invitrogen, Paisley, U.K.). Proteins were separated by SDS/PAGE and transferred (20 volts, overnight, 4 °C) onto PVDF membranes (Millipore, Watford, U.K.) immersed in transfer buffer (25 mM Tris base, 192 mM glycine, 0.5% [w/v] SDS, 17% [v/v] methanol). Non-specific antibody binding was minimised by incubating membranes with blocking buffer (10% [w/v] BSA, 20 mM Tris base, 137 mM NaCl, 0.1% [v/v] Tween-20, pH = 7.6) for 1 hour at RT. Some membranes were cut into two parts, which were probed separately with different primary antibodies for 2 hours at RT (1:1000), followed by incubation with HRP-conjugated secondary antibody (1:5000) for 1 hour at RT. Enhanced chemiluminescence (ECL) was used to detect protein bands with Amersham ECL Prime Western Blotting Detection Reagent and Amersham Hyperfilm ECL (G.E. Healthcare Life Sciences, Buckinghamshire, U.K.). Films were developed using a Fuji medical film processor FPM-100A (FUJIFILM U.K. Ltd., Bedford, U.K.). Different film exposure times were used for each blot and those generating the greatest contrast between the bands were selected for subsequent quantification.

Developed X-ray films were scanned using a Xerox WorkCentre 7855 photocopier (Settings: greyscale; default for brightness/sharpness/saturation; 100% zoom; 600 dpi) to generate JPEG image files. Unprocessed image files were opened into ImageJ (v1.50) and no adjustments (e.g., brightness/sharpness/saturation) were made. Protein bands were quantified as follows: identical areas (height (100) × width (150) = 15,000 pixels) were drawn around each protein band and the density of all pixels (scale 0–255 each) summed. The integrated density of each area was therefore quantified on a scale from 0 (totally white) to 3,825,000 (totally black). Values are presented as % black. Background density levels were determined (approx. 45–48% black), representing no protein signal. Data were not un-blinded until quantification of all blots was complete.

### 5-HT uptake into platelets

Platelets take up extracellular 5-HT via SERT and store it in dense granules. SERT activity was quantified by monitoring the reduction in extracellular concentration of 5-HT following the addition of 1 μM 5-HT to WP (2.00 × 10^8^ mL^−1^). After addition of 5-HT, aliquots of WP were removed at 5 min intervals, added to a STOP solution to minimise subsequent uptake, immediately centrifuged (13,000 rpm, 1 min, RT), and the supernatant frozen.

HPLC (see above) was used to separate and quantify 5-HT from WP supernatants. A reversed phase C18 column (Kinetex: 250 × 4.6 mm, 5 μm beads, 100 Å pore size) (Product code 00G-4601-E0, Phenomenex, Cheshire, U.K.) and isocratic method (mobile phase: 94% [v/v] phosphate buffer (18.4 mM citric acid, 83.2 mM K_2_HPO_4_; pH = 6.6) and 6% [v/v] acetonitrile) were used at 40 °C with a flow rate of 1 mL min^−1^. 5-HT appeared as a peak at 4.25 min, detectable at 276 nm. Chromatograms were analysed using N2000 Chromatography Data System (Tianjin University, China). 5-HT standards in CFT were measured on each experimental day to determine 5-HT concentrations in the WP supernatants.

### Lactate dehydrogenase cytotoxicity assay

Lactate dehydrogenase (LDH) release from WP was determined as an indicator for drug-induced cytotoxicity and cytolysis, using a Pierce LDH activity assay kit (Thermo Fisher Scientific, Loughborough, U.K.). WP (250 µL, 2.00 × 10^8^ mL^−1^) were treated with citalopram for 10 min, supernatants isolated following centrifugation (13,000 rpm, 1 min, RT) and 50 µL aliquoted into wells of an Immulon-2HB 96-well flat-bottom plate. 50 µL of the proprietary reaction mixture (Thermo Fisher, product code: 1862887) was added to each well for 30 min. 50 µL of the proprietary stop solution (Thermo Fisher, product code: 1862880) terminated the reaction, and background absorbance at 680 nm was subtracted from the absorbance at 490 nm. Measurements were taken using a Tecan Sunrise absorbance microplate reader (Tecan U.K., Reading, U.K.).

### Data modelling and statistical analysis

Concentration-response data were modelled using a four-parameter logistic (4PL) model^60,63^:

1 $${R}_{PRED}=\frac{Min-Max}{1+{([A]/{10}^{-p{A}_{50}})}^{{n}_{H}}}+Max$$

where: *R*_*PRED*_ = predicted response (dependent variable); [*A*] = agent concentration (independent variable); *Min* = response when [*A*] = 0; *Max* = response when [*A*] = ∞; *pA*_50_ = −log [*A*] (expressed in units of mol.L^−1^) when *R*_*PRED*_ = (*Max* + *Min*)/2; *n*_*H*_ = Hill coefficient. When *A* is an inhibitor, the *pA*_50_ is the *pIC*_50_.

For 5-HT uptake experiments, the decrease in 5-HT concentration was modelled using a first order kinetic model to derive rate constants for 5-HT uptake:

2 $${C}_{t}={C}_{0}{e}^{-{k}_{u}t}$$

where: *C*_*t*_ = [5-HT] (µM) at time *t* (min); *t* = time from addition of 5-HT (min); *C*_0_ = [5-HT] (µM) when *t* = 0; *k*_*u*_ = rate constant for 5-HT uptake (min^−1^). The rate constant represents the probability of 5-HT uptake per unit time and is therefore a direct measure of levels of active SERT.

Unless otherwise stated, fitting was performed using minimisation of least squares with the Solver function in Microsoft Excel. Data are presented as mean ± standard error (SE) unless otherwise stated. ANOVAs were performed using the UNIANOVA procedure in IBM SPSS (v23). Figures were generated using R (v3.3.2) (The R Foundation for Statistical Computing, Vienna, Austria).

Densitometry data were fitted to the 4PL model, with additional parameters incorporated to model basal levels of phosphorylation (i.e., no agonist) and background (no protein). Non-linear mixed effects modelling (densitometry and 5-HT uptake data) was performed using NONMEM 7.3 (Icon PLC, Dublin). The objective function used by NONMEM 7.3 was extended least squares, and is determined using maximum likelihood estimation^64^. NONMEM allows data from all experiments to be analysed simultaneously and random variation between experiments to be incorporated and quantified. This eliminates the requirement for data normalisation, generates more precise population parameter estimates, and allows specific hypothesis tests to be performed between alternative models using likelihood ratio tests (LRT)^65^.

### Data availability statement

The datasets generated and analysed during the current study are available on request.

## Electronic supplementary material

Supplementary methods

Supplementary Data
