# Supplementary material for: Citalopram inhibits platelet function independently of SERT-mediated 5-HT transport
Source: Sci Rep. 2018 Feb 22;8:3494. doi: 10.1038/s41598-018-21348-3 (PMC5823918; doi:10.1038/s41598-018-21348-3)
Supplement: Supplementary file 1 — Supplementary methods [file 41598_2018_21348_MOESM1_ESM.pdf]

Citalopram inhibits platelet function independently of SERT-mediated 5-HT transport

Harvey G. Roweth[1], Ruoling Yan[1], Nader H. Bedwani[1], Alisha Chauhan[1], Nicole Fowler[1], Alice H. Watson[1], Jean-Daniel Malcor[2], Stewart O. Sage[1] and *Gavin E. Jarvis[1]

Supplementary Methods

Table S1: Constituents and Gradients of Mobile Phases for HPLC Methods

Method	Mobile Phase A	Mobile Phase B	Gradient Time (min)	(A:B)
Nucleotide detection	Phosphate buffer<br>2.2 mM K_2HPO_4;<br>47.8 mM KH_2PO_4;<br>pH = 5.45	Acetonitrile	0.00	(100:0) (sample injection)
			1.50	(100:0)
			1.51	(98.5:1.5)
			2.70	(98.5:1.5)
			4.20	(91.0:9.0)
			5.70	(91.0:9.0)
			5.80	(25:75)
			6.80	(75:25)
			6.81	(100:0)
5-HT detection	Phosphate buffer<br>18.4 mM citric acid;<br>83.2 mM K_2HPO_4;<br>pH = 6.6	Acetonitrile	0.00	(94:6) (isocratic method)
