# Supplementary material for: Citalopram inhibits platelet function independently of SERT-mediated 5-HT transport
Source: Sci Rep. 2018 Feb 22;8:3494. doi: 10.1038/s41598-018-21348-3 (PMC5823918; doi:10.1038/s41598-018-21348-3)
Supplement: Supplementary file 2 — Supplementary Data [file 41598_2018_21348_MOESM2_ESM.pdf]

Citalopram inhibits platelet function independently of SERT-mediated 5-HT transport

Harvey G. Roweth[1], Ruoling Yan[1], Nader H. Bedwani[1], Alisha Chauhan[1], Nicole Fowler[1], Alice H. Watson[1], Jean-Daniel Malcor[2], Stewart O. Sage[1] and *Gavin E. Jarvis[1]

Supplementary data showing western blot films generated during the study

Supplementary Figure S1

Donor: A

* Blot ID: HGR_039

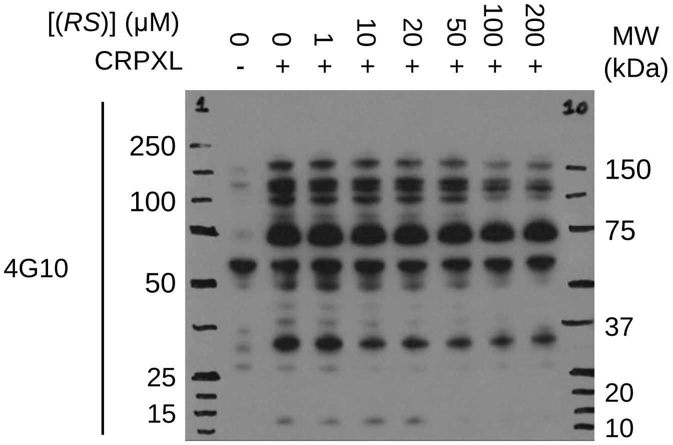

Donor: F

Blot ID: HGR_020

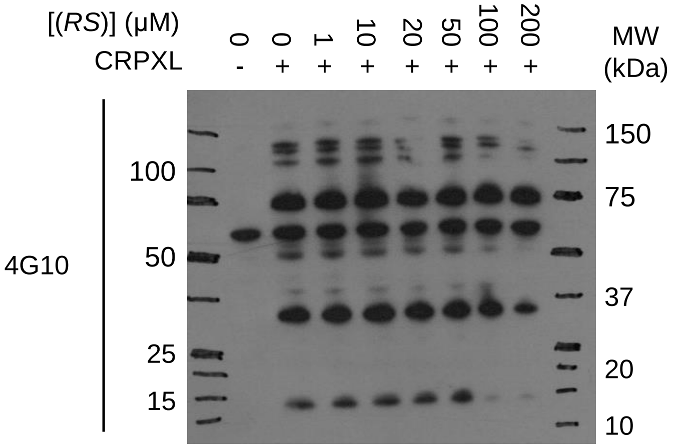

Supplementary Figure S1 (continued)

Donor: E

* Blot ID: HGR_037

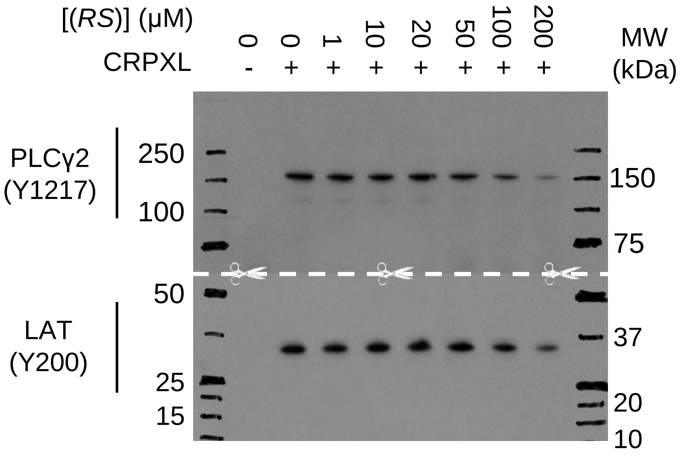

Blot ID: HGR_038

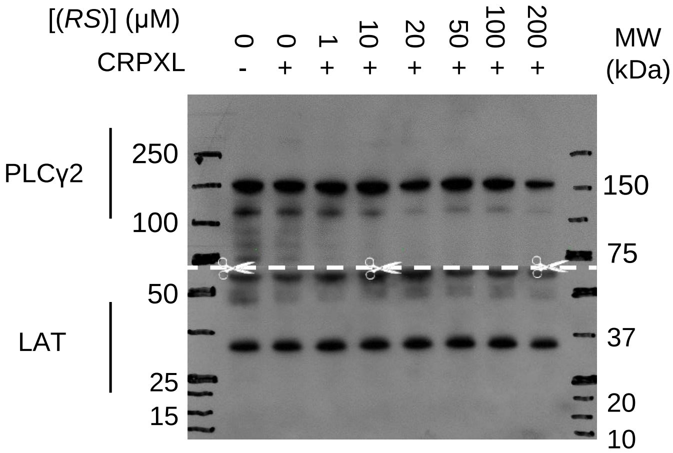

Donor: E

* Blot ID: HGR_062

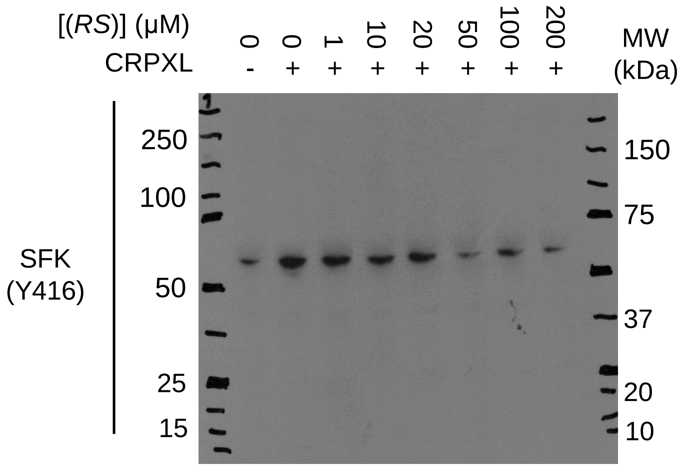

Blot ID: HGR_063

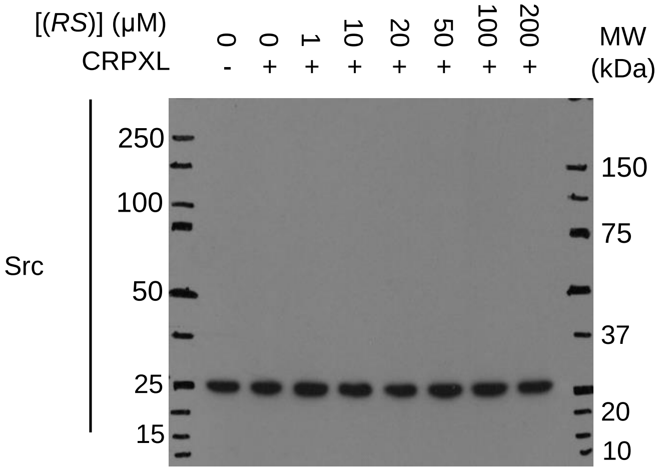

Supplementary Figure S1 – Pilot study data

Unprocessed images of X-ray films of western blots generated in a pilot study. Samples were probed with primary antibodies against phospho-tyrosine (4G10) (N = 2 donors), and phospho-PLCγ2 (Y1217), phospho-LAT (Y200), phospho-Src family kinases (SFK) (Y416), PLCγ2, LAT and Src (N = 1 donor). Films shown in the main manuscript are indicated with *. For undetermined reasons, the Src blot (HGR_063, Donor E) produced a band at 25 kDa, despite Src having a molecular weight of ~60 kDa. All subsequent Src blots (Supplementary Figure S3) show bands at ~60 kDa. None of these images or any pilot study data were used in the quantitative analysis shown in Fig. 5b. 3 blood donors were used in total, labelled A, E and F.

Supplementary Figure S2

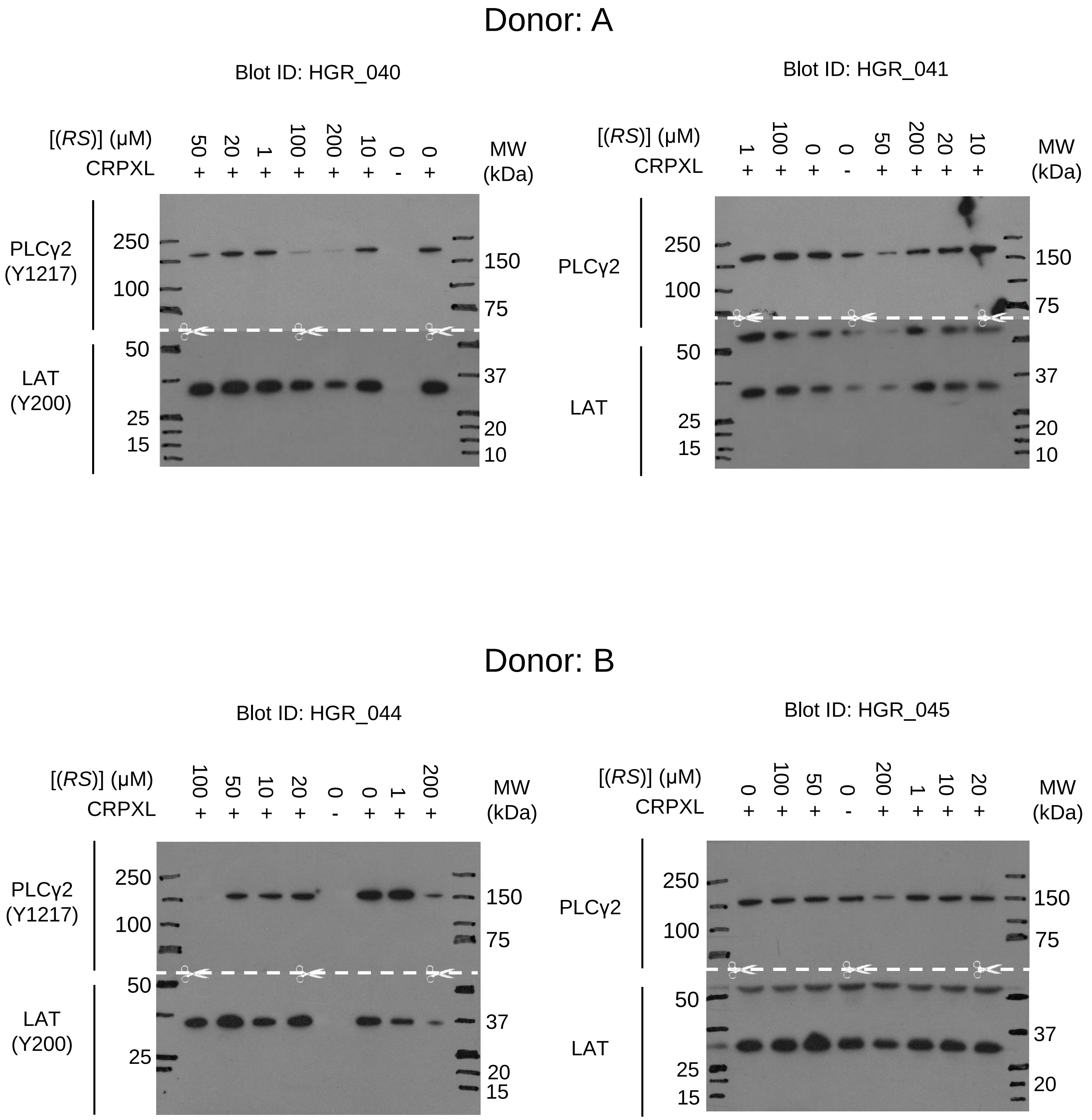

Supplementary Figure S2 (continued)

Donor: C

Blot ID: HGR_046

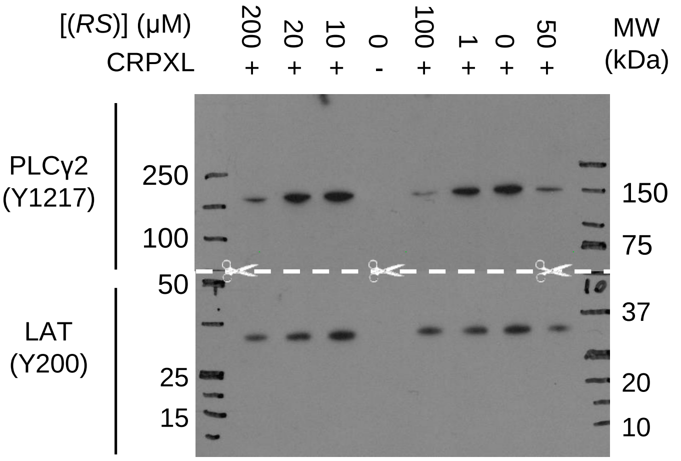

Blot ID: HGR_047

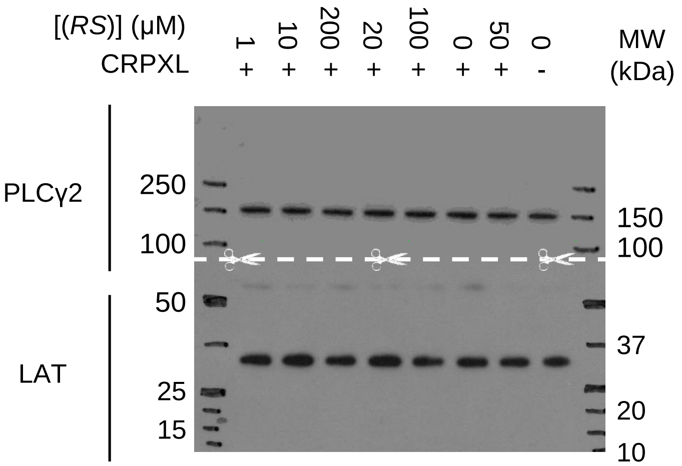

Donor: D

Blot ID: HGR_048

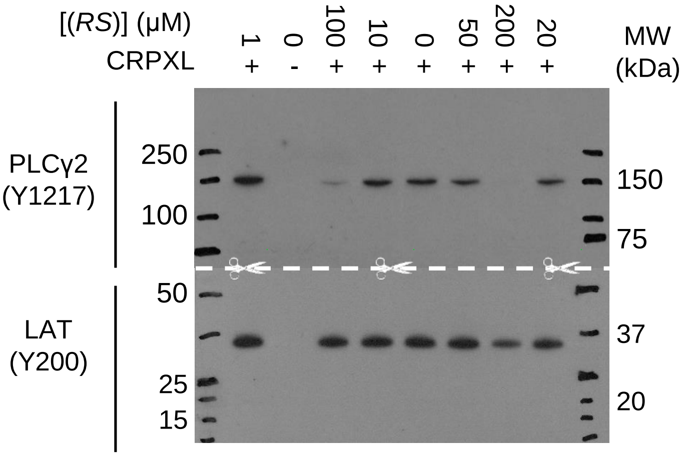

Blot ID: HGR_049

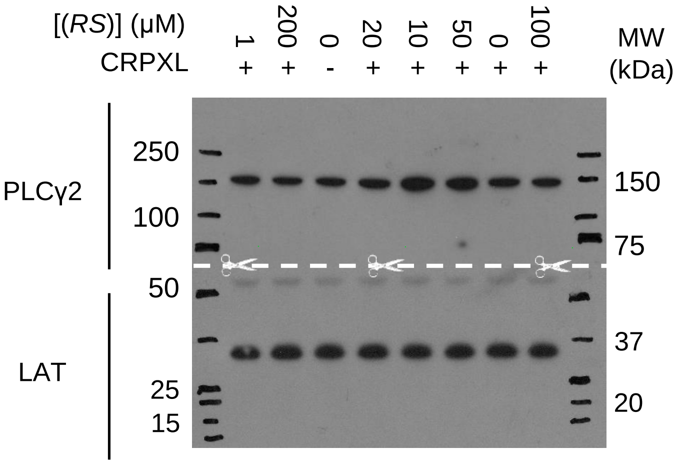

Supplementary Figure S2 (continued)

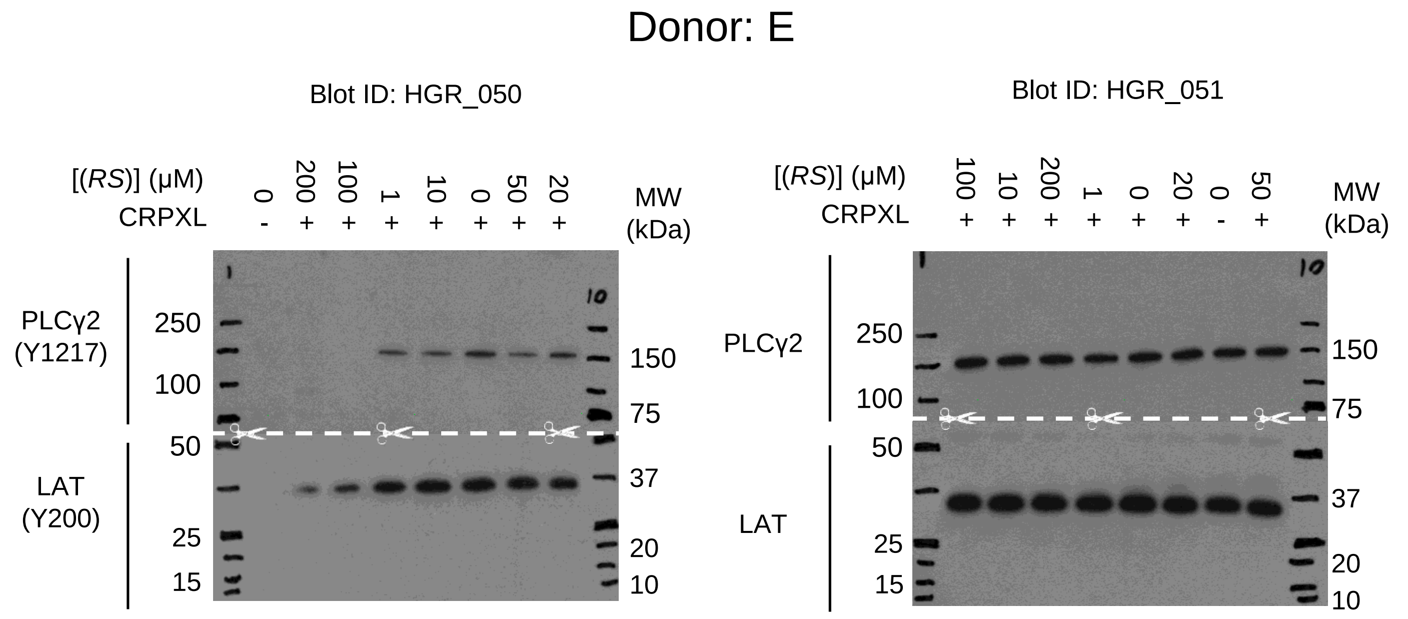

Supplementary Figure S2

Unprocessed images of X-ray films of western blots used for the quantification of phospho-PLCγ2 (Y1217), phospho-LAT (Y200), PLCγ2, and LAT. 5 blood donors were used in total, labelled A-E. White scissors and dashed white lines indicate where membranes were cut for subsequent blotting with different antibodies for either PLC or LAT. Samples were randomised and allocated into wells 2-9 in a blinded fashion.

Supplementary Figure S3

Donor: A

Blot ID: HGR_070

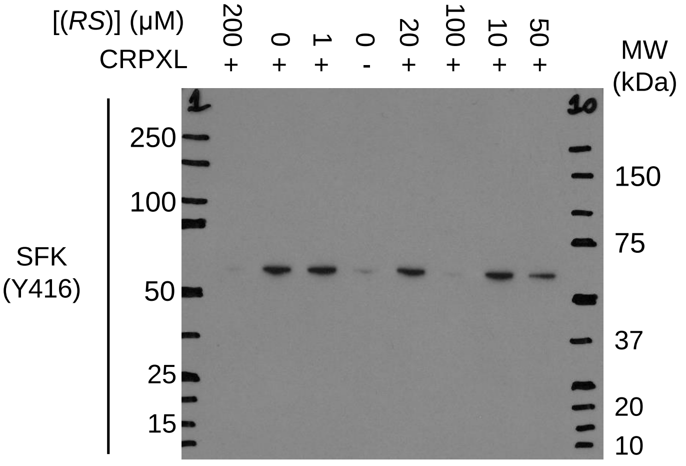

Blot ID: HGR_071

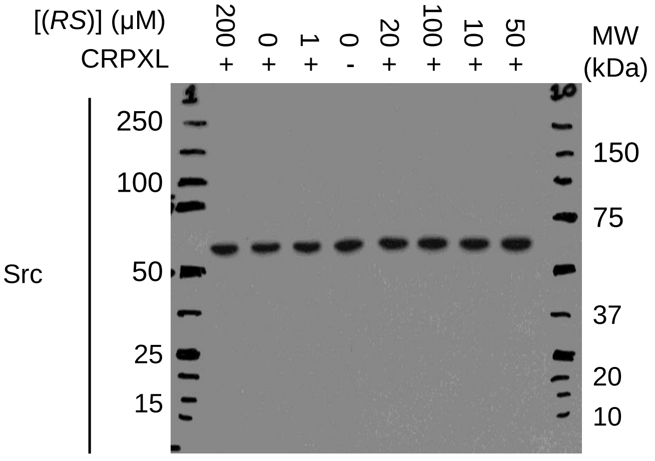

Donor: B

Blot ID: HGR_068

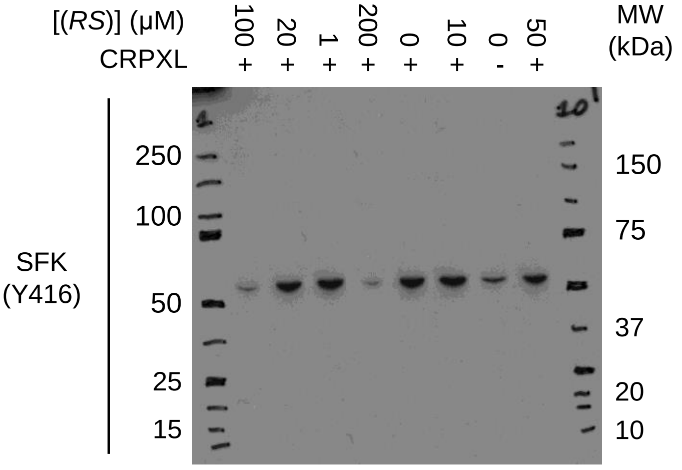

Blot ID: HGR_069

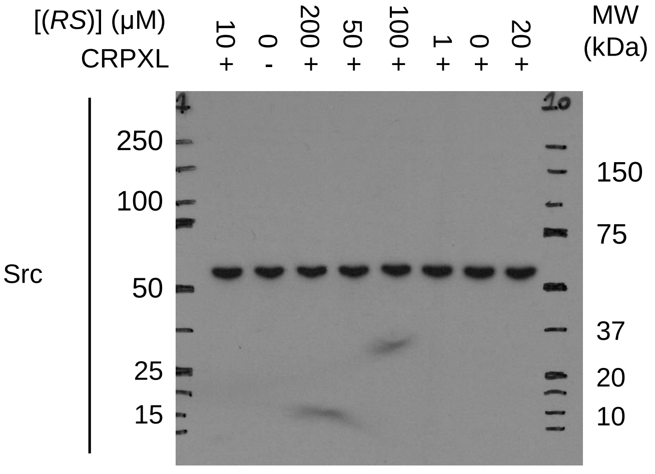

Supplementary Figure S3 (continued)

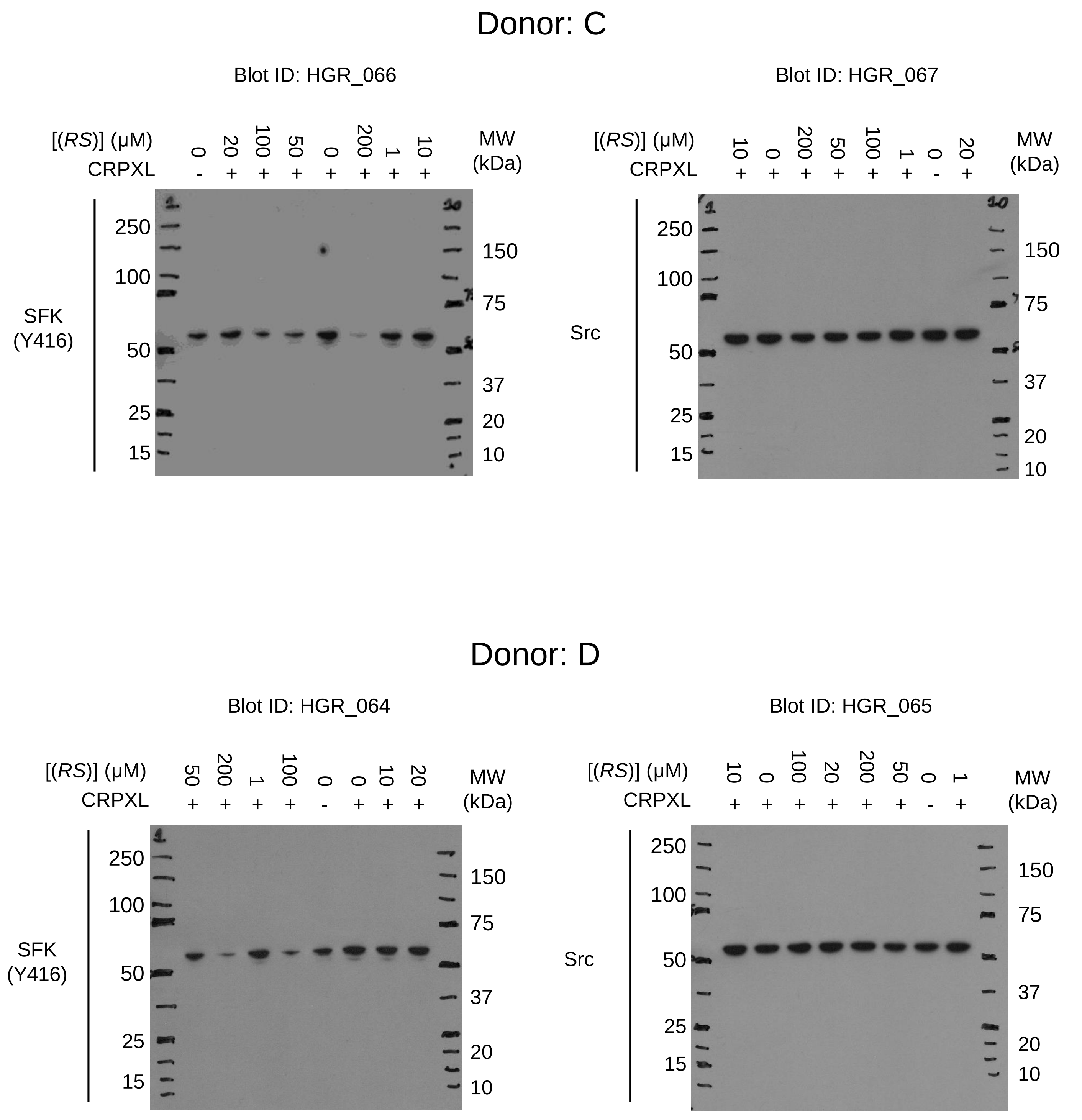

Supplementary Figure S3

Unprocessed images of X-ray films from western blots used to quantify phospho-Src family kinases (SFK) (Y416) and Src. Samples from 4 blood donors were used in total, labelled A-D, which match donors used in Supplementary Figure S2. No data were obtained for donor E as there was insufficient material from this donor for all conditions when the analysis was performed. Samples were randomised and allocated into wells 2-9 in a blinded fashion.
